# Pathways to recovery model of youth substance misuse in Assam, India

**DOI:** 10.1111/hex.13658

**Published:** 2022-11-09

**Authors:** Anna Madill, Raginie Duara, Sangeeta Goswami, Rebecca Graber, Siobhan Hugh‐Jones

**Affiliations:** ^1^ School of Psychology University of Leeds Leeds UK; ^2^ Institute of Positive Mental Health & Research Guwahati Assam India; ^3^ School of Humanities and Social Science University of Brighton Brighton East Sussex UK

**Keywords:** addiction recovery, LMIC, mental health, substance abuse disorder, visual methods

## Abstract

**Introduction:**

There are global calls for better understanding of substance use disorder (SUD) to inform prevention, risk reduction and treatment of this relapse‐prone disorder. Our aim in this article is to understand the pathways to recovery of youth in Assam, India who have suffered SUD.

**Methods:**

We recruited 15 participants (11 men and 4 women) via two rehabilitation facilities. All are addicts‐in‐recovery aged 19–24 years. Material was generated through photo‐led interviews, analysed using an inductive variant of thematic analysis and the resulting model refined through expert and participant checks.

**Results:**

We present a multiroute, multidirectional pathway to recovery model. It has three phases, *Recreational Use*, *Addiction* (*Relaxed*, *Chaotic*, *Strategic*) and *Supported Recovery*, each phase consisting of cycling between, or transitioning through, a series of stages.

**Conclusions:**

The model enhances psycho‐socio‐cultural insights into the experience of risk and recovery, and informs prevention and treatment for youth substance misuse in Assam. This is the first model of its kind and an important public health resource. We discuss the possible transferability of the model to a wider range of contexts.

**Patient or Public Contribution:**

The model presented was generated through analysis of interviews with addicts‐in‐recovery. Four of these addicts‐in‐recovery, and two mental health and rehabilitation service providers, conducted participant and expert checks of the model leading to its improvement.

## 1 INTRODUCTION

There are global calls for better understanding of substance use disorder (SUD) to inform prevention, risk reduction and treatment of this relapse‐prone disorder.^1^ Global mental health strategies, in particular, prioritize adolescents given the high prevalence of SUDs in this population, trajectory towards lifelong disadvantage and suicide risk.^2^ Similarly, the India Mental Health Survey 2015–2016^3^ calls for the strengthening of youth mental health research and addiction management. Subsequently, the first government‐commissioned national survey was conducted in 2019^4^ on the extent and pattern of substance use in India. Recommendations include legal and policy innovations, scaled‐up treatment programmes and prevention interventions targeting young people.

At 14.6% of the Indian population, alcohol is the most commonly used psychoactive substance, predominating in men at 17:1, with about 19% of users deemed dependent. Cannabis (2.8%) and opioids (2.1%) are the next commonly used substances. About 0.25% of cannabis users showed dependency and 0.70% of all Indians need help with opioid use. About 0.20% of all Indians abuse sedatives, while inhalant abuse is higher in children and adolescents (1.17%) than adults (0.58%). A relatively minor problem is reported with regard to cocaine, amphetamine and hallucinogens. Earlier studies provide additional information on gender and youth. Gururaj et al.^3^ report that SUD is prevalent in 22.4% of the Indian adult population and is 2.5 times more common in men than women. Thirteen percent of those abusing substances are children and adolescents,^5^ with only 5% of those under 20 years old seeking treatment: the lowest by far of all age ranges.^6^ Finally, excluding tobacco, the most common substances abused by young people are alcohol, cannabis and opioids, with initiation typically between 13 and 15 years.^7^

In the late 1980s, the National Institute of Social Defense (NISD) identified nongovernmental organizations (NGOs) with expertise in substance abuse prevention and treatment and conferred them status as Regional Resource Training Centres (RRTCs) with a remit to provide training and monitor treatment centres.^8^ Twelve RRTCs exist and over 450 affiliated addiction treatment centres. De‐Addiction Centres or Drug Treatment Centres are also available in many psychiatry departments of government medical colleges and district and general hospitals where free medications may be dispensed.^9^ There are also numerous private treatment facilities. Hence, India has a comprehensive SUD treatment programme which includes ‘detoxification, pharmacotherapy, individual therapy, family therapy, group therapy, multifamily therapy, 12‐Step programs’.^10^ Programmes also integrate Indian cultural practices such as yoga, spirituality and an emphasis on social interdependence including support groups for families. However, more research is required into adapting the 12 steps for the Indian context,^11^ and rehabilitation facilities are usually single‐sex, with women underserved and disproportionately stigmatized.^12^

Defining recovery from SUD is contentious and increasingly described as a process rather than an event.^13^ Although many heterogeneous models of addiction exist,^14^ there is little research on the journey from drug initiation through to recovery that is not biologically based or of known relevance to young people, women and to Low‐and‐Middle‐Income‐Countries (LMIC). In the present article, we describe the typical routes through SUD to recovery as narrated by young Indian people who have walked this path. Listening to young people and developing services *for* them *with* them is deemed best practice in global health.^15^ However, this is a relatively novel approach to informing mental health services and policy in India where the value of evidence from service users and young people can be underestimated.^16^

Assam is a state in northeast India. It is geo‐politically isolated, has been propelled into the 21st century from an agrarian social base and the needs of children, youth and women requires urgent attention.^17^ A high stake is placed on scholastic achievement and material affluence, while structural inequalities and lack of opportunity to develop life skills contribute to college dropout, suicide and SUD. In fact, the Assam State Report of the National Mental Health Survey of India identifies adolescent SUD as an urgent public health problem.^18^

It is difficult to find statistics on adolescent SUD in Assam, particularly by gender. Hazarika et al.^19^ report that of 10–19 years old (*N* = 63) living in a border area, 4.8% used alcohol and 3.2% (only males), used drugs such as heroin and solvents. Islam et al.^20^ report that 80% of street children in Guwahati aged 5–18 years (*N* = 215) abused substances, 87.4% of whom abused solvents. Katoki et al.^5^ report substance abuse amongst adolescents from the urban slums of Guwahati (*N* = 60) to predominate in males at a ratio of 4:1, with the highest rates of abuse between 16 and 19 years. A worrying level of solvent abuse and young initiation age typically of 8–13 years in Assam is also noted by Priyanka and Ankita.^21^ Recommendations of the Assam State Report of the National Mental Health Survey include working closely with rehabilitation services, decreasing stigma and encouraging help‐seeking through better public awareness.

Our study focuses on 19–24 years old who have suffered SUD but have not used addictive substances for at least 1 year. A sustained sobriety definition of recovery was chosen because it is the target of the rehabilitation services in Assam with whom we were working. Moreover, as a signatory of the United Nation's International Conventions (Article 47), India is obligated to ‘act to *eliminate* the use of illicit drugs, to develop measures to prevent drug use and to ensure availability of treatment for people with drug use disorders’.(^3^,p.689, italics added) Hence, the aim of this study is to understand the pathways to recovery of youth in Assam who have suffered SUD. In so doing, we seek to enhance psycho‐socio‐cultural insights into the experience of risk and recovery, and inform prevention and treatment for youth SUD in the region.

## 2 METHOD

Approval was obtained from the Ethics Committee of the LGB Regional Institute of Mental Health, Assam and from the Ethics Committee of the School of Psychology, University of Leeds, UK.

### 2.1 Recruitment and participants

Recruitment was undertaken by two of our Partner Organizations (POs) in Assam: Nirmaan Rehabilitation Facility and Hope Foundation Rehabilitation Centre. These organizations have direct contact with the demographic of interest and are widely networked throughout the rehabilitation, voluntary and educational sectors. Both are private rehabilitation facilities, charge a fee for care, and, as is typical, serve only male clients. Nirmaan Rehabilitation Facility has 16 staff, all trained in counselling and therapy, and a visiting psychiatrist. It follows the 12‐step programme along with spiritual principles and offers 90‐day residential treatment. Hope Foundation Rehabilitation Centre is a satellite of a global charity and also follows the 12‐step programme. It has 14 trained staff and a part‐time psychiatrist. Their work includes detoxification, rehabilitation and an extended care unit for those who have completed their 90–120‐day course.

Candidates for the study had to be Indian nationals, aged 19–24 years, in successful recovery from drug and/or alcohol abuse (i.e., 1 year substance‐free, irrespective of tobacco use). Most participants had daily contact with our POs, some living within the premises while giving service, with records kept of client progress. Hence, clean time was assessed through face‐to‐face contact with counsellors and through the extended service user network dedicated to providing close support. Other participants, including all the women, were identified by our POs through their service user network, including Alcoholic Anonymous and Narcotics Anonymous meetings. One of these women recovered with peer as opposed to professional support. These networks are based on ‘good faith.’ However, recovery networks overlap substantially with personal life and people ‘know each other's business’ to a much greater extent that in many western contexts and our POs did not recommend for the study three women and five men they were monitoring because they deemed them to have relapsed before the requisite 12 months.

The Betty Ford Institute Consensus Panel^22^ identifies three timeframes of recovery associated with increasing resilience to relapse: early recovery (1–12 months), sustained recovery (1–5 years) and stable recovery (5+ years). Hence, in this categorization, our participants have entered sustained recovery. We did not include tobacco‐only users given its relative social acceptance and because the Assam State Report is of the opinion that ‘(t)obacco use per se, is not an issue for mental disorders’.(^17^, p.18).

In line with Dworkin's^23^ recommended sample size for this method of research, we set a minimum target of 12 participants and, given the preponderance of men with SUD, aimed for a sample of approximately three‐quarters male. Hence, we commenced with a purposive sampling strategy with regard to gender. As recruitment continued, we monitored for diversity across our age range of interest and substance of addiction. Heroin was the main substance of addiction for eight participants, alcohol for five and weed and cocaine for one each. Other drugs used, as described by participants, are brown sugar, cannabis, cough syrup, inhalants, marijuana, tablets and uppers. Nine participants were working as rehabilitation service providers, three in another form of employment, two were students and one was unemployed. Saturation of key themes and concepts for the men occurred after approximately six interviews and key differences in the women's accounts as compared to the men's were being reiterated in the interview with the fourth and final female participant.^24^

Our POs identified candidates from their service user communities, by word of mouth, and by distributing information about the study within their networks. Interested candidates recommended to us by our POs were provided the information sheet and invited to discuss the study in a face‐to‐face meeting where the procedure and conditions of consent were explained in the candidates’ preferred language. If suitable, and wishing to take part, candidates were provided guidance material on collecting images to bring to interview (see Supporting Information). Detailed guidance was provided, such as the suggestion that ‘You could start by thinking about the most important issues (or times, events, or people, or experiences) that you would like to talk about in the interview and then find an image, or take a photograph, that represents this in some way. The image can be of the thing itself or it can symbolize it’.

### 2.2 Data collection

Audio‐recorded photo‐led interviews were conducted between April 2019 and October 2020 at the premises of our POs, including Mind India: a private registered society operating throughout northeast India which provides counselling, psychosocial interventions and training. To enhance anonymity, participants were asked to provide verbal consent only which was audio‐recorded before interview. Interviews were conducted in a mixture of Assamese and English. After the interview, consent was rechecked based on the participant now knowing what they had disclosed.

The interview topic guide (see Supporting Information) was developed in consultation with team members with expertise in rehabilitation and counselling in Assam. Interviews commenced with background information such as current employment situation. The usual format was then to ask the participant, ‘Is there a picture you would like to share first?’ using prompts where appropriate such as, ‘What were your relationships with other people like at this point in your life?’ and the interview ended with a request for feedback on the process of collecting images. Interviews lasted between 55 and 235 min (mean = 114 min) and the number of images brought ranged from 7 to 33 (mean = 12).

Analysis was conducted iteratively with data collection and queries raised through the analysis fed‐back into the interview process. In practice, this did not change the interview topic guide but helped us identify where it was useful to add prompts to elicit more information if needed, for example about current daily functioning. Interviews were transcribed in English verbatim with translations from Assamese and checked for accuracy by two members of the team.

### 2.3 Data analysis

We used an inductive variant of thematic analysis.^25^ Each transcript was assigned to two researchers who read it carefully and made general notes on the participant's recovery narrative. The assigned pair then discussed observations in an online meeting, one taking notes on agreed phenomena of interest, tentative patterns, concepts and themes, questions raised by the analysis and provided a short summary of the participant's story. These notes were passed to the second researcher for revision until agreed upon. The team met online together several times throughout the process of analysis to discuss the observations being made. As analysis progressed, the team decided to focus on patterns in the trajectory of the participants' stories of recovery having observed similar strategies and cycles in the material. We then rotated schematics between us until a pathways model was agreed upon and then credibility was checked. Hence, for the analysis reported here, our specific thematic analytic approach is: (i) a detailed account of one particular aspect of the data set; (ii) inductive as opposed to theory‐driven; (iii) content‐driven as opposed to interpretative and (iv) takes a realist as opposed to constructionist epistemological stance.

**Figure 1:**
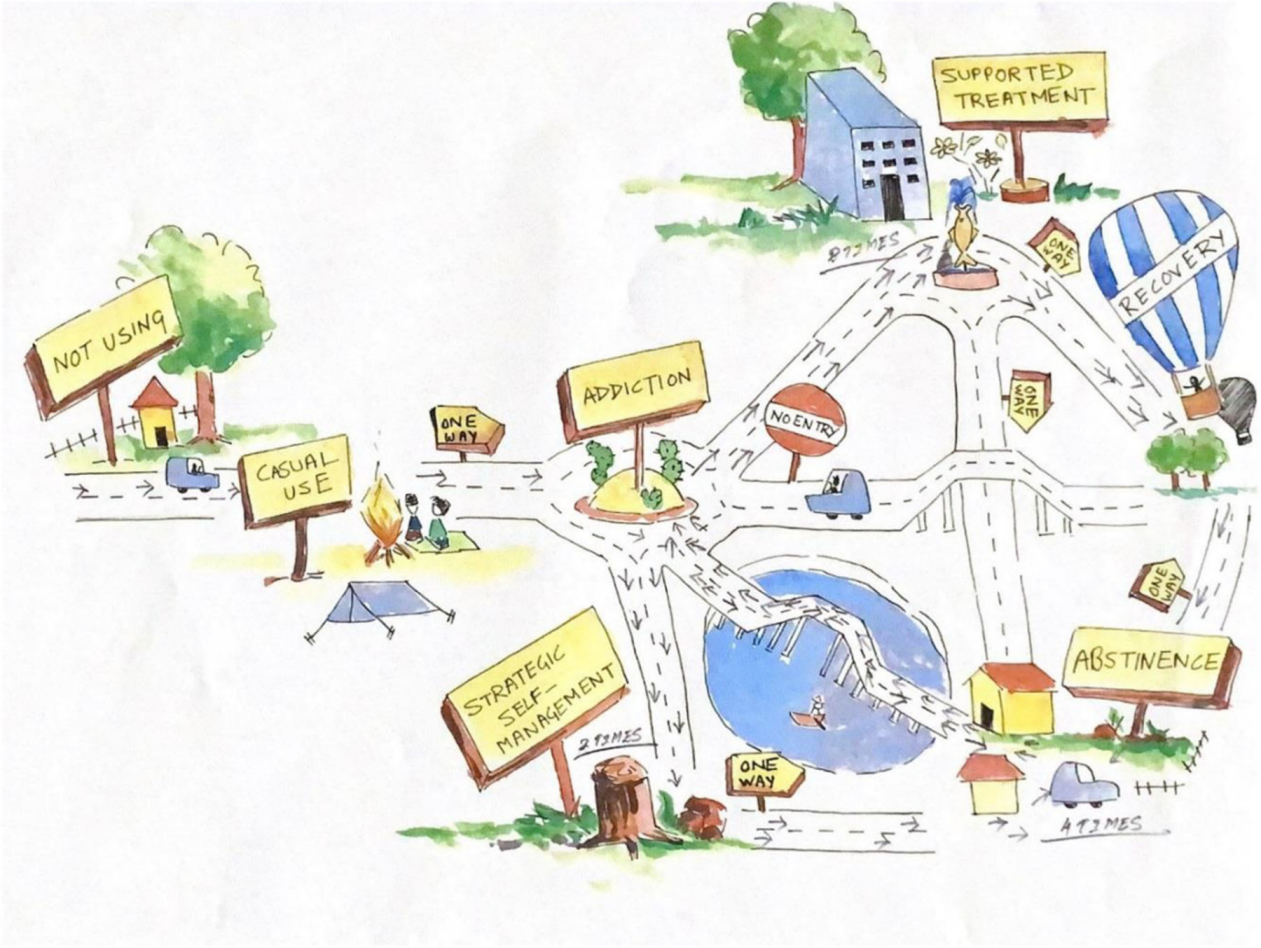
One man's pathway to recovery. Noncommercial license https://licensing.leeds.ac.uk/product/pathways-to-recovery-model-of-substance-use-disorder-youthassam-comics-type-images-and-animations.

We present a pathways to recovery model that made sense of the complexities of the participants' narratives. This model was refined through expert and participant checks. An expert check was conducted with a clinical psychologist at LGB Regional Institute of Mental Health, Assam and with a senior addictions counsellor at Nirmaan Rehabilitation Facility. The expert check led to: (i) clearer justification of terminologies for, and articulation of, the stages *meaningful treatment* and *strategic self‐management* and, (ii) an additional pathway from *strategic self‐management* to *abstinence*. The senior addictions counsellor at nirmaan rehabilitation facility discussed the model further with members of staff at the rehabilitation facility. This resulted in adding a one‐way path from *meaningful treatment* to *abstinence* to recognize that necessary lifestyle changes may not be evident after treatment even if substance use has ceased. Four participants (two men; two women, including the woman who recovered through peer support) who were willing to contribute further then individually took part in checking the model. The model was presented in pictorial form to aid understanding and each asked if they could use it to track their own journey to recovery (Figure 1). Each was able to do so with relative ease, with one (see Supporting Information) suggesting that the path between *in recovery* and *abstinence* should be two‐way because there can be a period during which substances are not being abused but the recovery lifestyle is collapsing.

## 3 RESULTS

We identify three phases defined as characteristic ways of relating to addictive substances: *Recreational Use*, *Addiction* (*Relaxed*, *Chaotic* and *Strategic*) and *Supported Recovery* (Table 1). Each phase consists of a cycle between, or transition through, a series of stages. *Recreational Use* cycles between the stages of *not using* and *casual use*. *Relaxed Addiction* is a unique subphase consisting of a particular nonchalant attitude to the stage *in addiction*. The subphase *Chaotic Addiction* cycles between the stages of *in addiction* and *abstinence*, while *Strategic Addiction* cycles between the stages of *in addiction* and *strategic self‐management*. The phase *Supported Recovery* consists of a transition through the stage *meaningful treatment* and settles on the stage *in recovery*, but may involve many cycles and relapses into different subphases of *Addiction*. The final element of our model, *transitions*, refers to the movement between stages each of which has a more *positive* or *negative* valance with regard to progress towards recovery.

**Table 1:** Phases, stages and transitions on the pathways to recovery model

Phases	Phase 1 Recreational Use		Phase 2 Addiction							Phase 3 Supported Recovery				
			Phase 2a Relaxed Addiction	Phase 2b Chaotic Addiction			Phase 2c Strategic Addiction							
Stages	Stage 1 Not using		Stage 3a In addiction	Stage 3a In addiction			Stage 3a In addiction			Stage 3a In addiction				
	Stage 2 Casual use			Stage 3b Abstinence			Stage 3c Strategic self‐management			Stage 4 Supported treatment				
										Stage 5 In recovery				
Transitions and valence	1 Into causal use	2 Into quitting casual use	3 Into addiction	4 Into abstinence	5 Into relapse	6 Into recovery	7 In‐and‐out of strategic treatment	8 Into abstinence	9 Into accepting support to quit	10 Into relapse before being in recovery	11 Into abstinence before being in recovery	12 Into recovery	13 Into relapse after being in recovery	14 Into abstinence after being in recovery
	−ve	+ve	−ve ‘event horizon’	Fragile +ve	−ve	+ve (rare)	−ve	+ve (rare)	+ve	−ve	Fragile	+ve	−ve	−ve

We now describe the phases, stages and transitions in more detail and provide evidence through quotes from across our participants. The symbol […] is used to indicate a small portion of text omitted within quote and we indicated if a quote is from a female participant.

### 3.1 Phase 1: Recreational Use

**Image 1:**
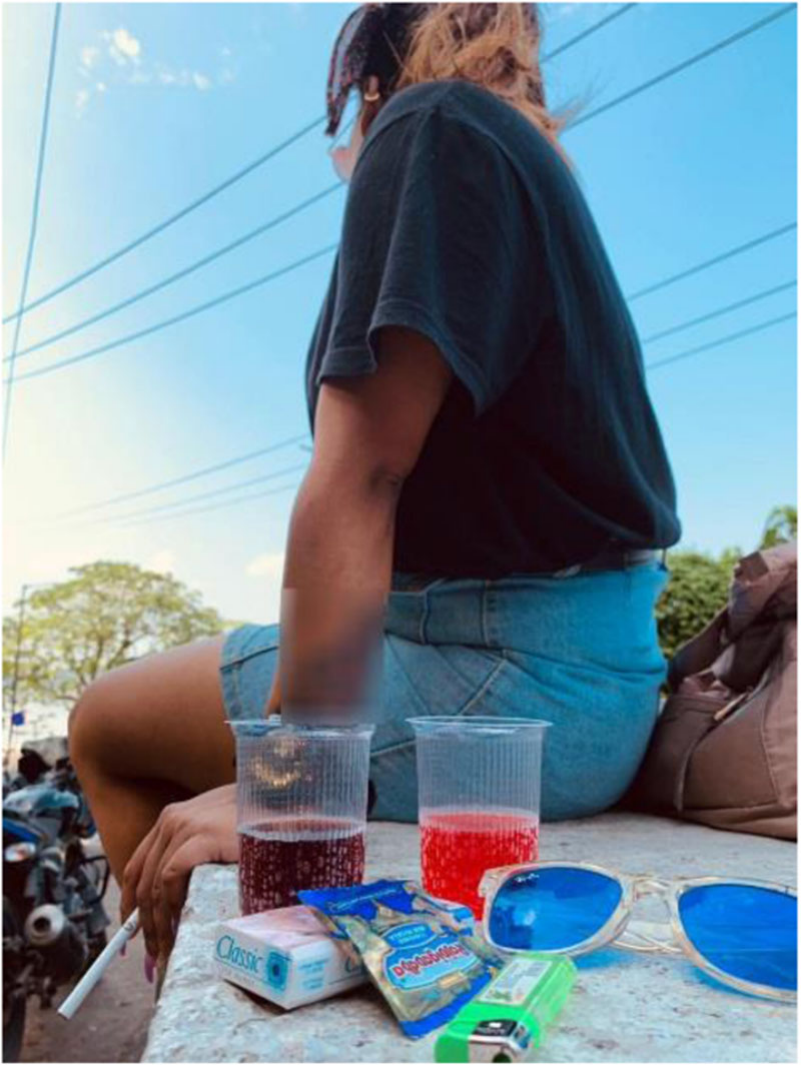
One photograph brought by a female participant

All participants engaged with addictive substances at the beginning for one, or a combination, of the following psychosocial reasons. First, some wanted to gain credibility with older peers: ‘They didn't call anybody else, only me. I went and they were drinking there. They offered. I did drink too’. Second, others just wanted to join‐in with friends: ‘the boys who live in my neighbourhood they too use it. And they told me “We will have alcohol. Will you have?”’ Third, curiosity was a key motivation: ‘my curiosity to know about it was increasing so then they made it by mixing with cigarette and gave me’ (female). Finally, some got into the *Recreational Use* of substances because they wanted to escape boring and/or difficult life circumstances: ‘Mom always showed her sorrows in front of me. Today we don't have this much money. […] I am staying good and I am getting the crisis. I will intoxicate [refers to Image 1]’ (female).

In the phase of *Recreational Use*, participants had the opportunity to cycle between the stage of *not using* and the stage of *casual use*. However, it often was not apparent that a transition had occurred into the stage *in addiction* until there was difficulty securing the needed substance(s). As one female participant explained, continual opportunities to use substances may obscure the fact that addiction has occurred: ‘often there is party so our withdrawal doesn't happen’ (female). However, at this point the ability to be a ‘take it or leave it’ *casual user* is no longer possible: ‘I tried leaving but means the symptoms started showing’.

### 3.2 Phase 2: Addiction (Relaxed, Chaotic and Strategic)

The phase of *Addiction* is reached via a transition constituting a *negative* ‘event horizon’ from which there is no return to *Recreational Use*. *Addiction* has three distinct, possible subphases all of which share the stage *in addiction* but are inflected with different subjective experiences and behaviours. There may be cycling between the three subphases, although they have a logical progression through a subjective orientation of nonchalance, to distress, to destructive adaptation.

In the subphase *Relaxed Addiction*, needed substances are largely available and, because the positives are perceived generally to outweigh the negatives, there is little or no subjective experience of distress or motivation to quit. For example, as one participant explains: ‘my main problem was fear, social anxiety, social awkwardness. That thing was completely removed by alcohol and cannabis for a temporary time but I thought that it is a permanent solution’. Depending on circumstances, *Relaxed Addiction* can become chronic or is a transitory lull: ‘I lie around drinking alcohol. If I think about it, useless. Means what will I do by thinking?’

The likely next subphase is *Chaotic Addiction* which cycles between the stages of *in addiction* and *abstinence*. During this subphase, there are periods of not using because the needed substance(s) cannot be accessed or because there is an attempt to quit on one's own: ‘Even ma'am [teacher] got to know that I was drunk. She got the smell. I sit near her. I felt very bad and I then I quit for some days. Means I didn't have it. Then my head started aching in the morning and little means I started wanting it. Keeps giving me cravings. Then it all started again’.

Our model does provide for the possibility that *abstinence* may lead to the stage *in recovery*, and one of our female participants did indeed take this route. However, without external support the transition to *abstinence* is fragile and may lead to relapse back to *in addiction*, especially for young adults. A key reason for relapse is that no personal or lifestyle changes have been made: ‘I've stayed clean for six months. So what happened to me? A misunderstanding developed between me and my family. I too had misbehaved’. Commensurate with this position, the female participant who recovered without professional help, did attend Alcoholics Anonymous and received mentoring from an addict‐in‐recovery. Moreover, the main gender difference we found was the way in which women needed to consider the implications of using professional support due to the possibility of being too identifiable as an (ex‐)addict: ‘my mother, grandmother and to everyone said that. “Why is it required to let her go to rehab? She will not be able to get married. Who is going to marry her?”’ (female).

The likely next subphase is *Strategic Addiction* in which there is a cycling between the stages of *in addiction* and *strategic self‐management*. The essential feature of *strategic self‐management* is that there is no real or sustained intention to quit. Instead, interventions are engaged with to mitigate negative impacts and to sustain the addiction. Interventions may be used to mollify other people: ‘it was like it's better for me to stay in a rehab for like two three months. Be there and not use drugs. Gain my trust back from my family […] and then after that when I am out I will find new ways to get money, drugs’. Interventions may also be engaged to manage physical symptoms in the short term and deal with temporary interruptions to supply. One participant illustrates this in relation to opioid substitution therapy (OST): ‘the day I don't get money from home I take OST then this was my mentality. Family doesn't get to know. My addiction is also sustained’. A particularly common form of *strategic self‐management* is short‐term, medically supervised, family financed detoxification undertaken often on multiple occasions: ‘I started making excuses about all that too that I'll do detox. I need money for medicine. Give me money […] with that money I again keep taking heroin’.

### 3.3 Phase 3: Supported Recovery

In the phase *Supported Recovery*, the stage of *in addiction* moves into that of *meaningful treatment* and subsequently, if fortunate, to *in recovery*. Central to the transition from *in addiction* to *meaningful treatment* is acceptance of the support needed to quit. For example, one participant reflected on his experience of reaching out almost despite himself: ‘Don't know what happened. I phoned that day to that counsellor. Phoned him that day and just asked how he was. He asked “What are you doing?” I said “What would I do? I'm doing substances.” He talked nicely and he talked so nicely that I thought means I should try once again’. There must also be a real and sustained intention and commitment to the personal and lifestyle changes entailed: ‘If I see someone that okay he is using she is using it's her personal life. I avoid that because I understood what are my priorities, what are the things I left back, what are the mistakes I had made in my life’ (female).

**Image 2:**
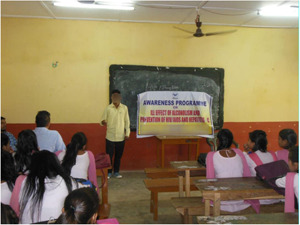
One photograph brought by a male participant

Despite engaging with *meaningful treatment*, there is the possibility of relapsing back to *in addiction* before being *in recovery* or after a period of being *in recovery*, directly or via *abstinence* defined as merely ‘not using’ without commitment to the long term: ‘After coming back from the centre, at home go out with friends. Same activity in my life. No change in the activity […] Life is not on track. Things which usually I won't do I do. So after that ah I had again’. The stage *in recovery* is always a work‐in‐progress and not an end‐point or ‘cure’ that has been reached once and for all: ‘I've even seen people who have had 15 years of clean time but they relapse […] we are real‐life soldiers who are always fighting for our lives’. Hence the importance of continued contact with a recovery community: ‘they are my seniors also and they are my using friends and we all are in recovery right now together […] basically I found my family here’. Some also sustained their recovery through the meaning they found in supporting others: ‘when they called I agreed. I said “OK I will do awareness programme. I want to [refers to Image 2]”’.

## 4 DISCUSSION

We offer a multiroute, multidirectional pathway to recovery model of youth substance misuse in Assam. Three studies have been conducted on the pathways to care of adult men in the context of SUD in New Delhi. These indicate a delay in treatment‐seeking and report family, friends and neighbours as the main sources of encouragement.^26^, ^27^, ^28^ Although important, knowledge of pathways to care does not tell us about the preceding journey into, and through, addiction, nor the subsequent, often long and convoluted, path to recovery. Moreover, there is little or no information on the pathways of women or young people with SUD, or in India outside New Delhi.

In a very different nonwestern context, Fatayir^29^ (cited in Ali et al.^30^) offers a model of the trajectory through addiction from an Arabian perspective. Five stages are proposed: *Discovery* of pleasure; *Honeymoon* of consolidated consumption; *Early Addiction* where the substance is prioritized; *Elevation of Addiction* of personal and social deterioration; and finally *Zenith of Addiction* in which everything is destroyed except focus on the substance. There are interesting parallels with our model suggesting some potential transferability to wider contexts. *Discovery* may be similar to *Recreational Use*; *Honeymoon* to the transition to, and sojourn in, *Relaxed Addiction; Elevation of Addiction* may echo *Chaotic Addiction;* and *Zenith of Addiction* echo *Strategic Addiction*. However, unlike our model, Fatayir^29^ does not include recovery, implies a linear trajectory based on the intensity of focus on the substance, and may have been developed from men's experiences only. Moreover, our model allows for individualized and cycling trajectories around a small number of behaviourally recognizable phases with differential intervention needs.

In a western context, the Transtheoretical Model of Change (TTM) is a popular addiction recovery framework.^31^ First of five stages is *Precontemplation* in which no problem is acknowledged. Second is *Contemplation* where some thought is given to the problem and what to do. Third, in the *Preparation* stage, there is an intention to change. The fourth stage is *Action* in which change is initiated. Finally, in the *Maintenance* stage, efforts are made to consolidate progress and avoid relapse. Comparing TTM with our own model, youth in *Recreational Use* and *Relaxed Addiction* are likely *Precontemplators*. Interestingly, those in *Chaotic* and in *Strategic Addiction* are already in *Action*, presumably having undergone some *Contemplation* and *Preparation*. However, they have initiated action that undermines recovery, inadvertently in *Chaotic Addiction* and deliberately in *Strategic Addiction*. For the most part, it is only those in *Supported Recovery* that have taken effective *Action* and have a chance of reaching *Maintenance*.

Research is mixed on the efficacy of TTM for understanding addiction and recovery, and a major criticism is that the stages are arbitrary and overlap. However, as is the case with our own model, DiClemente^31^ does not claim that TTM posits a determined order or single linear pathway but is, instead, a developmental model of recovery.^32^ On the other hand, TTM is a generic model of change and does not appear to capture well the cycling, cynical and self‐destructive processes of addiction, particularly those we identify as *Chaotic* and *Strategic*. Moreover, TTM may posit an overly rational and individualistic change processes contrary to the often distorted and socially situated experiences informing our model. However, models like TTM, with a strong theory of change may complement inductively derived, contextually situated models such as ours and contribute to the transferability of our work to wider contexts (see also Hansen et al.^33^).

In Phase 1 of our model, *Recreational Use*, the risk of engaging with addictive substances is experienced as psychosocial pushes and/or pulls. Pushes consist of attempts to escape boring and/or difficult life circumstances. Pulls consist of personal curiosity and the need for social prestige and integration with peers. Similarly, a study of nursing students in Karnataka,^34^ found the main causes of substance abuse to be, in order of frequency, peer pressure, enjoyment and family problems. In Assam, itself, Katoki et al.^5^ report slum‐living adolescents to be primarily influenced to take illicit substances by friends and peers and, secondarily, out of enjoyment or curiosity. Arguably, difficult life circumstances are endemic to this group, and fights, vandalism and criminal activities were reported by Katoki et al.^5^ alongside SUD. These psychosocial pushes and pulls can be extremely powerful and we can extrapolate with support from the literature that resilience to drug initiation will include the presence of positive peer influences and role models, and strong family and community support,^35^ particularly parental monitoring.^36^ To this we add the directing of curiosity in constructive directions.

In Phase 2 *Addiction*, a major risk is remaining in an extended period of *Relaxed Addiction* because substances are not perceived to be a problem and/or are viewed as a remedy to pressing psychosocial issues. Although opinion is somewhat divided in the literature,^37^ our participants were clear that, in their experience, having entered *Addiction*, it is impossible to return to a ‘take‐it‐or‐leave‐it’ *Recreational Use* pattern. This may be due to biological changes^38^ and/or the troubled contexts in which substances are secured and consumed.^39^ Illicit drug use is often attempted self‐medication, particularly in relation to psychological disorders. However, as Temmingh^40^ points out, there is little research on this in LMIC. Psychosocial skills education, and early identification and treatment of young people with psychological disorders, could prevent many cases of SUD.^41^

Although research indicates that it is possible to recover from SUD unassisted, for example, through ‘maturing out’,^42^ our work demonstrates that a risk for young Assamese people attempting to quit substances alone is entering an extended period of *Chaotic Addiction* characterized by a cycle of *abstinence* and relapse. A key aspect of this phase is captured by the slogan ‘abstinence is not recovery’, in which recovery is considered to be a ‘voluntarily maintained lifestyle characterized by sobriety, personal health and citizenship’.^35^,p.259 In the context of another nonwestern county—South Africa—Stokes et al.^43^ report also the importance of a psychological mind‐set for sustaining recovery, avoiding situations associated with substance use, and keeping otherwise meaningfully engaged. The skills and stamina to transform abstinence into recovery are unlikely without a strong, nonjudgemental support system of people and organizations that know what it takes.^44^ However, as evidenced by one of our female participants, support does not need to be via official services.

Finally, within *Addiction*, there is a risk of settling into *Strategic Addiction* in which there is no real or sustained intention to quit but only to mollify other people and/or to deal on a known temporary basis with negative impacts and interrupted supply. In fact, a key finding of our study is that many parents and service providers are unaware of, or that a young person is in, *Strategic Addiction* and so waste scarce resources supporting interventions that actually sustain addiction. Specifically, our study suggests that, in Assam, there may be a particular risk of inadvertently colluding with *Strategic Addiction* through placing too much emphasis, and hope, on stand‐alone detoxification treatment.

Important to understanding Phase 3 *Supported Recovery* is that it comprises, indefinitely, a complex constellation of de‐addiction support, meaning that recovery must be viewed as a continual work‐in‐progress. Stokes et al.^43^ report the importance of social support while adding that a transition into recovery is often sparked by a crisis turning‐point. In Assam, people with SUD and associated mental health challenges are often stigmatized and assumed to be criminals.^45^ As an educational tool, our model has the potential to challenge myths about SUD and increase the possibility of constructive community support for young people and their families. The transformation of community ‘gossip’ from negative to positive over an individual's recovery journey is documented in rural America, supporting the conclusion that community education is an important route to help addicts into long‐term recovery.^46^ Although our participants were predominantly from urban settings, there are strong resonances with Krentzman and Glass's^46^ study given tendency to the interweaving of lives in Assamese neighbourhoods. There is also potential to explore longer‐term recovery as Webb et al.^47^ did with a British sample to understand the transferability of their findings that gratitude and reliance on support groups transformed into greater self‐determination and independent decision‐making.

It is exceptionally important to recognize the phase and stage in which a young person is so that the most appropriate intervention can be made. The two transitions associated with Phase 1 *Recreational Use* offer particularly fruitful points for *prevention* interventions: (i) prevention of starting casual drug use and (ii) prevention of SUD through quitting casual drug use before addiction occurs. The risk of slipping into addiction unawares cannot be overestimated and is noted also in Hansen et al.'s^33^ study of nine American addicts in long‐term recovery. However, prevention interventions are not appropriate if the young person has, even unwittingly, entered the phase of *Addiction* and that this has happened is often hidden deliberately from others.

Phase 2 *Addiction* interventions are best geared towards *accepting support to quit* and the importance of admitting to needing help is highlighted also by Hansen et al.^33^ The process of recovery is difficult to sustain on one's own and *Chaotic Addiction*, in which this is attempted, can be physically, psychologically and socially destructive and lead to a dangerous level of hopelessness. Medicalised interventions with no long‐term psychosocial follow‐up are particularly problematic and can be used cynically in *Strategic Addiction*. Although it is a major success to enter Phase 3 *Supported Recovery* there is always a risk that commitment to recovery flounders, and relapse can occur before or after a period of being in recovery. Support from others is vital to develop a real and sustained commitment to the personal and lifestyle changes required, particularly in peer recovery‐oriented communities.^35^

We incorporated a purposive sampling strategy, selecting for diversity to generate rich and relevant material for an in‐depth study. However, the relatively small sample size and situational specificity could be viewed as limitations. For example, to recruit 15 participants, 27 people attended an initial meeting to discuss the study and, although Hope Foundation Rehabilitation Centre identified five candidates, only one took part. We do not know why individuals did not join the study and the information sheet stated that we would not ask. Informally, we understand that several candidates relapsed just before reaching a substance‐free year. All but one of our participants had attended residential rehabilitation services and, hence, are distinguished by having access to some financial resources. Moreover, 9 of our 15 participants were working as service providers in the recovery sector. However, many rehabilitation facilities in Assam provide the opportunity to give service as part of on‐going recovery and Stokes et al.^43^ note that helping others and working in a recovery environment is common for addicts in sustained recovery.

In terms of strengths, our participants were reasonably representative in terms of the type of addictive substances engaged, including those most commonly abused by young people in India, that is, alcohol (*N* = 14), cannabis (*N* = 11) and opioids (*N* = 7)^7^ and, in Assam, solvent abuse (*N* = 5).^20^ Alcohol was the main addiction of three of the four women, but this is commensurate with national figures that 26.3% of women aged 15–49 years in Assam consume alcohol, the highest by far of the 36 states surveyed.^48^ Finally, our model was confirmed in expert and participant credibility checks including both male and female addicts‐in‐recovery and the participant who did not use professional rehabilitation services.

The key implications of this research for policy development in Assam are as follows. In addiction, interventions are best geared towards encouraging a young person to accept support to quit. Effective interventions, including medical treatment, require also long‐term psychosocial support to have the best chance of sustaining sobriety. Investment in women's rehabilitation is needed due to the immense stigma women experience, even when in recovery, and will have the additional potential benefit of contributing to the well‐being of their current and future children. Finally, investment in family and community education and peer‐to‐peer support is likely an economical and effective strategy for preventing youth SUD and enabling rehabilitation.

In support of these potential policy initiatives, we have cocreated a visually informed community education package around our model.^49^ Early piloting has been conducted in Assam with high school students, the general public, postgraduate mental health trainees and women in rural and semirural districts. This demonstrated that the education package was successful in promoting young people's voice with regard to SUD prevention and recovery, increasing awareness of their needs and has the potential for stigma reduction. We are currently demonstrating our educational package to schools, colleges, rehabilitation services and health providers in Assam to encourage uptake, collect feedback and cocreate further ideas for incorporating the model in activities such as group lesson plans, personal recovery journaling and peer‐to‐peer mentoring. Future research includes a trial of the effectiveness of the education package and extending our understanding of the pathways to long‐term recovery from SUD in Assam.

## AUTHOR CONTRIBUTIONS

**Anna Madill**: Conceptualization (lead); formal analysis (lead); funding acquisition (lead); methodology (lead); project administration (lead); resources (equal); supervision (lead); visualization (equal); writing – original draft preparation (lead). **Raginie Duara**: Conceptualization (supporting); data curation (lead); formal analysis (supporting); investigation (lead); methodology (supporting); project administration (supporting); visualization (equal); validation (lead); writing – original draft preparation (supporting). **Sangeeta Goswami**: Conceptualization (supporting); formal analysis (supporting); investigation (equal); methodology (supporting); project administration (supporting); resources (equal); validation (supporting); writing – review & editing (equal). **Rebecca Graber**: Conceptualization (supporting); formal analysis (supporting); funding acquisition (supporting); methodology (supporting); writing – review & editing (equal). **Siobhan Hugh‐Jones**: Conceptualization (supporting); formal analysis (supporting); funding acquisition (supporting); methodology (supporting); writing – review & editing (equal).

## CONFLICT OF INTEREST

The authors declare no conflict of interest.

## ETHICS STATEMENT

Approval was obtained from the Ethics Committee of the Lokopriya Gopinath Bordoloi Regional Institute of Mental Health, Assam and from the Ethics Committee of the School of Psychology, University of Leeds, UK. The study conforms to the Declaration of Helsinki guidelines. All persons gave their free informed consent before their inclusion in the study.

## Supporting information

Supplementary information.

## Data Availability

The data that support the findings of this study are openly available in ReShare at https://reshare.ukdataservice.ac.uk/855418/.
