# Supplementary material for: Pathways to recovery model of youth substance misuse in Assam, India
Source: Health Expect. 2022 Nov 9;26(1):318–28. doi: 10.1111/hex.13658 (PMC9854309; doi:10.1111/hex.13658)

Pathways to recovery model of youth substance misuse in Assam, India

Photovoice Guidance

Thank you for taking part in this research. 'Photovoice' is the use of photographs or images to help people to tell their story.

1. Getting started with Photovoice

We would like you to bring between **7 and 10** photographs or images to the interview which will help us understand:

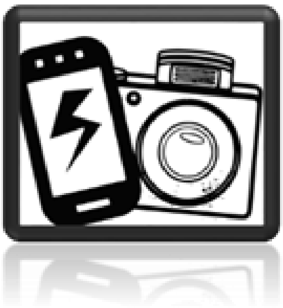

What it has been like for you resisting drugs/alcohol.

OR

What it has been like for you recovering from drug/alcohol problems.

You could start by thinking about **the most important issues** (or times, events, or people, or experiences) that you would like to talk about in the interview and then find an image, or take a photograph, that represents this in some way. The image can be of the thing itself or it can symbolize it. For example, you may want to talk about the feeling that you have few options sometimes, so you could for example take a picture of a menu. If you wanted to explain the importance of time and space away from problems, you could take a picture of a quiet place that matters to you.

You could use the time between now and the interview **to notice things** in your life that you think are important to this research topic and take a picture of things as you go along. For example, you might be playing sport and realise that this really helps you - so you might take a picture of a football. You might also look through **past photographs** and this might trigger your memory about times, places, events or people that might help us understand your experience. You could choose one or two that will remind you in the interview of the kinds of issues you wanted to talk about. You can take a picture o f a picture or a photo if that saves you having to print anything out.

Photovoice recognizes that an image is not the whole story, and that words sometimes aren't enough, but together the images and our interview will help give a fuller story of what things have been like for you.

2. Important boundaries

We recommend to avoid taking new photos of other people unless they are known to you. However, if you would like to take photo of another person, that person (i) should be over the age of 18 and (ii) should be asked permission before including them in the photo you bring for the interview. Also, when other people are there, only take a photo if feel very comfortable that everyone will be OK about it. Please *do not* take

photographs of anyone under the age of 18. Instead, you can photograph something to represent them (e.g., a toy or a hoodie). Remember that if you bring an image of anything that makes the researcher concerned about your safety or the safety of others, or criminal activity, we may need to involve others who can help – but we will talk to you about this first. You can check the study information letter again for the details.

3. Photos for printing

Here are the options for sending photos to print.

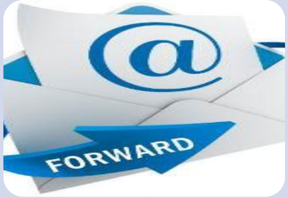

Click in your camera phone (or with the camera phone provided).

Email or Whatsapp them to me at least a day before the interview and I will print them out for us

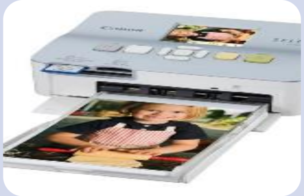

Bring the photos in your camera phone (or the camera phone provided to the interview).

Photos will be printed in a portable printer on the day of the interview.

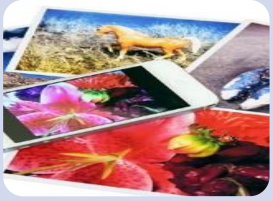

You can bring printed images that you already have.

I will take a photo of it so we have a record in our study.

We will conceal any identifying details of people or places in images before using them in a public way.

4. The interview

I will go through the consent process and ask you some general questions about yourself so that we can describe in reports the kind of people who took part (e.g., age range). Once you're ready, you will be asked to pick a picture that you would like to start with. I will listen to why that image is important to you and what you hoped it would convey. I may ask you more questions to understand your experience as best I can. Together we will move through each of the images in this way.

Feel free to get in touch if you have any questions along the way and I will help.

Interview Topic Guide (19-24 year olds)

Thank you for coming to this interview. I'd like to hear your story as far as possible in your own words. You sent me and have brought some pictures to help me understand your journey to recovery. Do you have any questions about the study before we start?

As we talk, please remember the following:

- try not to use people's names if you are talking about things they might not want other people to know (for example, they sometimes use drugs);
- be vague about dates and places to avoid it being too obvious who you might be talking about;
- a reportable criminal activity for us would be the selling but not the using of illegal drugs.

Before we start the interview, I would like to audio-record your consent. I will read out the conditions of consent and ask you to confirm each. You have a copy of these to keep. When we start the interview, I'll ask some basic information about yourself – but nothing that will make you identifiable. I want to remind you, though, that you don't have to answer any of my questions and can leave at any point.

I am starting to audio-record now. TAKE CONSENT Thank you. We will start the interview now.

Interview structure	Questions
**Demographics**	Do you mind telling me how old you are?<br>Are you currently employed? / What is it you do?<br>What three words best describe who you were before you started to tackle your substance use?<br>What three words best describe who you are today?<br>Thank you.
**Introduction**	Is there a picture that you would like to share first?
**Prompt**	Can you tell me about a bit about this image?
**The photographs**	Can you tell me what this picture means to you?<br>How does this link with your experience of substance use?<br>What does this image express about your journey to recovery?
**Prompts**	Which picture would you like to share next?<br>What were your worries at this point?<br>What difficulties were you facing here?<br>What responsibilities did you have at this point in your life?<br>What were your relationships with other people like at this point in your life?<br>How did you cope with that?<br>Do you have any regrets about that?
**Concluding questions**	Did other people understand what you were going through?<br>How are young people who use drugs and alcohol treated by other people?<br>Who makes these kinds of judgement? (peers?, friendly?, family?, teachers?)<br>What was most helpful to you during these difficult times?<br>How would you try and help a friend who had started using drugs or alcohol?<br>What advice would you give to those trying to tackle alcohol and drug use in young people?
**The process of taking photographs/selecting images**	How did you find the process of taking photographs and selecting images?<br>What were the positives?<br>What did you find difficult?<br>How could we improve the process?<br>Is there a photograph you wish you had taken but haven't?<br>Were you able to express everything you wanted in these images?
**Check-in**	Having shared your pictures how are you feeling now?

Illustrative example: One young man's pathway to recovery

We present a short case study to ground the conceptual model in an actual life story. This male participant is 22 years old. At the time of interview, he had been in recovery from heroin addiction for 13 months and assists others in their recovery journey. This case was selected because the participant provided us with insightful feedback on the model by testing if he could use it to trace his pathway to recovery, which he was able to do (see series of arrows on Figure 1) while, at the same time, illuminating a needed adjustment as described in the article.

This young man started using weed and, as his use became more regular, was introduced to heroin by neighbourhood friends. When his parents noticed his withdrawal symptoms, they took him to the doctor and he was given medication to suppress the unpleasant effects. He tried to quit heroin twice through medication, but without success: "*I couldn't get out of it. I will have doctor's medicine for two days, three days, and after those two, three days when I get a little better, when physical withdrawal little, slowly, slowly things stopped feeling good.*" With no change of attitude or routine, he quickly relapsed: "*My patience level was zero. I got intolerant about things. And the latest thing was that my lifestyle was totally messed up".* Back on heroin, his physical condition got so bad that his parents felt they had no choice but to send him to a rehabilitation facility. While there, he focused on the positive messages from experienced addicts-in-recovery: "*I go after people who were like me but today are living their life nicely. I started following those people's footprint, how they did the first initial years."*

After completing the programme, although he had been highly motivated to quit, after a short period of abstinence he started using again: "*After going out I have not got those things. Those empty spaces have not filled up where the society cannot accept me and those things somewhere keep me away from others. So after doing like this I used again where again the same circle was repeating*". During his time in rehab, he learned that it is even harder to stop using after a relapse. Hence, he was quick to realise the dangers, and after two-and-a-half months, returned to treatment: "*I just called them immediately saying that I have relapsed and they said "It's okay." [...] I told my family members as well. They said 'It's good. If you want to go, then go.'"*

Figure 1: One man’s pathway to recovery

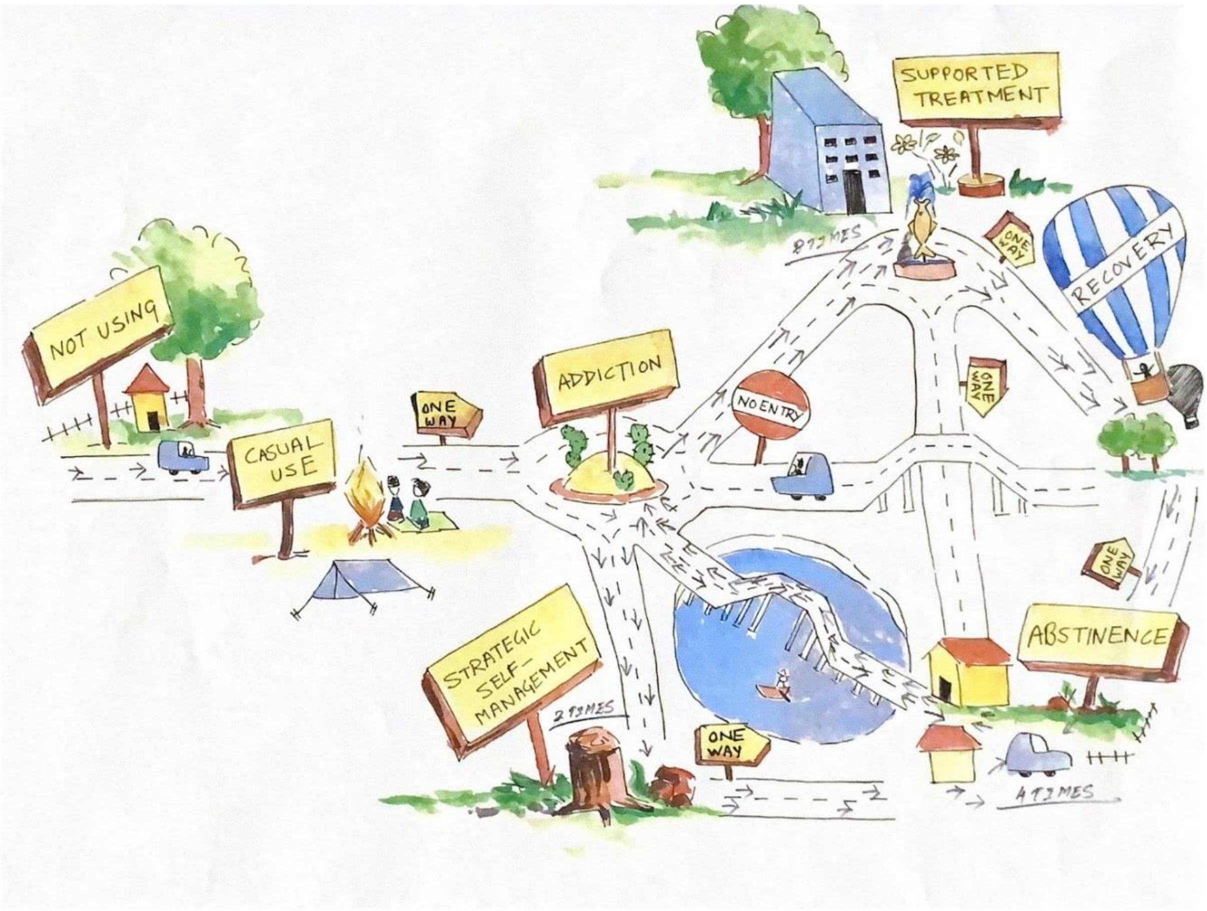

Supplement: Supplementary file 1 — Supplementary information. [file HEX-26--s001.pdf]
